# Reanalysing genomic data by normalized coverage values uncovers CNVs in bone marrow failure gene panels

**DOI:** 10.1038/s41525-019-0104-9

**Published:** 2019-12-09

**Authors:** Supanun Lauhasurayotin, Geoff D. Cuvelier, Robert J. Klaassen, Conrad V. Fernandez, Yves D. Pastore, Sharon Abish, Meera Rayar, MacGregor Steele, Lawrence Jardine, Vicky R. Breakey, Josee Brossard, Roona Sinha, Mariana Silva, Lisa Goodyear, Jeffrey H. Lipton, Bruno Michon, Catherine Corriveau-Bourque, Lillian Sung, Iren Shabanova, Hongbing Li, Bozana Zlateska, Santhosh Dhanraj, Michaela Cada, Stephen W. Scherer, Yigal Dror

**Affiliations:** 1 Genetics and Genome Biology Program, The Hospital for Sick Children, Toronto, ON Canada; 2 Marrow Failure and Myelodysplasia Program, Division of Hematology/Oncology, Department of Pediatrics, The Hospital for Sick Children, Toronto, ON Canada; 3 Pediatric Hematology-Oncology-Bone Marrow Transplantation, University of Manitoba, Cancer Care Manitoba, Winnipeg, MB Canada; 4 Department of Pediatrics, Children’s Hospital of Eastern Ontario, Ottawa, ON Canada; 5 Pediatric Hematology/Oncology, IWK Health Centre, Halifax, NS Canada; 6 CHU Sainte-Justine, Montreal, QC Canada; 7 Pediatric Hematology Oncology, Montreal Children’s Hospital, Montreal, QC Canada; 8 Division of Hematology/Oncology, UBC & B.C. Children’s Hospital, Vancouver, BC Canada; 9 Alberta Children’s Hospital, Calgary, Canada; 10 Children’s Hospital, London Health Sciences Centre, London, ON Canada; 11 Department of Pediatrics, McMaster University, Hamilton, ON Canada; 12 Centre hospitalier universitaire, Sherbrooke, QC Canada; 13 Royal University Hospital, Saskatoon, SK Canada; 14 Kingston General Hospital, Kingston, ON Canada; 15 Pediatric Hematology/Oncology, Janeway Child Health Centre, St. John’s, NF Canada; 16 Allogeneic Blood and Marrow Transplant Program, Princess Margaret Cancer Centre, University of Toronto, Toronto, ON Canada; 17 Centre Hospitalier Universitaire de Quebec, Sainte-Foy, QC Canada; 18 Pediatrics, University of Alberta, Edmonton, AB Canada; 19 Population Health Sciences, Research Institute, Division of Hematology/Oncology, Department of Pediatrics, The Hospital for Sick Children, Toronto, ON Canada; 20 Institute of Medical Science, University of Toronto, Toronto, ON Canada; 21 McLaughlin Centre and Department of Molecular Genetics, University of Toronto, Toronto, ON Canada

**Keywords:** Genetic testing, Medical genomics

## Abstract

Inherited bone marrow failure syndromes (IBMFSs) are genetically heterogeneous disorders with cytopenia. Many IBMFSs also feature physical malformations and an increased risk of cancer. Point mutations can be identified in about half of patients. Copy number variation (CNVs) have been reported; however, the frequency and spectrum of CNVs are unknown. Unfortunately, current genome-wide methods have major limitations since they may miss small CNVs or may have low sensitivity due to low read depths. Herein, we aimed to determine whether reanalysis of NGS panel data by normalized coverage value could identify CNVs and characterize them. To address this aim, DNA from IBMFS patients was analyzed by a NGS panel assay of known IBMFS genes. After analysis for point mutations, heterozygous and homozygous CNVs were searched by normalized read coverage ratios and specific thresholds. Of the 258 tested patients, 91 were found to have pathogenic point variants. NGS sample data from 165 patients without pathogenic point mutations were re-analyzed for CNVs; 10 patients were found to have deletions. Diamond Blackfan anemia genes most commonly exhibited heterozygous deletions, and included *RPS19*, *RPL11*, and *RPL5*. A diagnosis of *GATA2*-related disorder was made in a patient with myelodysplastic syndrome who was found to have a heterozygous *GATA2* deletion. Importantly, homozygous *FANCA* deletion were detected in a patient who could not be previously assigned a specific syndromic diagnosis. Lastly, we identified compound heterozygousity for deletions and pathogenic point variants in *RBM8A* and *PARN* genes. All deletions were validated by orthogonal methods. We conclude that careful analysis of normalized coverage values can detect CNVs in NGS panels and should be considered as a standard practice prior to do further investigations.

## Introduction

Inherited bone marrow failure syndromes (IBMFSs) are a group of genetically heterogenoeuous disorders with impaired production of one or more blood cell types. They usually present during childhood and are frequently associated with physical malformations and a high risk of myelodysplastic syndrome (MDS), leukemia, and other types of cancer.^1,2^ Over the last several decades mutations in over 100 genes have been reported to cause IBMFSs involving fundamental cellular pathways such as DNA repair, telomere maintenance, and ribosome biogenesis.^3–5^ Thus far, in all patients with IBMFSs and an identified genotype, mutations in one gene have been found sufficient to result in a given phenotype. In our recently published data, 59% of the patients with classified IBMFSs and 18% of the patients with unclassified IBMFSs were found to have point mutations using a NGS gene panel assay of about 70 known IBMFS genes.^6^

Copy number variants (CNVs are submicroscopic deletion or duplications of DNA stretches ranging from several hundreds base pairs to about 3 mb.^7^ They are common causes of inherited diseases,^8–10^ and have been recognized as an important cause of IBMFSs,^10^ for example, Fanconi anemia^11,12^ and Diamond Blackfan anemia.^13,14^ We previously found that 16.4% of the IBMFS patients without identified point mutations had pathogenic CNVs by single nucleotide polymorphism arrays or comparative genomic hybridization arrays.^3^ Unfortunately, all current methods to detect CNVs suffer from major limitations. Metaphase cytogenetics can only detect variants greater than 3 mb and may miss abnormalities in areas that are not well visualized. Furthermore, the exact size of CNVs and whether the cytogenetic abnormality affects the copy number of a specific gene cannot be determined by metaphase cytogenetics. Comparative genomic hybridization arrays and single nucleotide polymorphism arrays only detect CNVs that are larger than 100 and 10–50 kb, respectively. Some important genes may not be covered by these arrays due to difficulties in designing proper probes. Last, small indels can be missed by Sanger sequencing if the primers are designed to anneal to the normal sequence of the indel region. Whole genome sequencing may overcome some of the above limitation;^15^ however, currently the rate of CNV detection by whole genome sequencing is only 35–50%.

Since next generation sequencing (NGS) gene panel assays are increasingly used to identify point mutations in clinical and research laboratories,^6,16,17^ it would be of a great advantage if they can also be used to detect CNVs. Several previous studies suggested that CNVs can be inferred from NGS data.^18,19^ Importantly, Anders and Huber^20^ compared high-throughput sequencing data by several different methods using a negative binomial distribution and found that the normalized coverage ratio can control type I error.

The aim of this study was to determine whether CNVs can be detected in data from NGS gene panel assays using methods that compare nucleotide read depth in test samples to normalized control coverage values. We also aimed to characterize the identified CNVs and to evaluate their clinical relevance. To address these aims we studied a large number of patients with IBMFSs by an IBMFS NGS gene panel assay.

## Results

### Characteristics of the patients

Two hundred and fifty eight IBMFS patients without a diagnostic genotype were analyzed by the IBMFS NGS gene panel assay. Fifty three percent were male. At time of analysis, 138 patients had a specific syndromic diagnosis and 120 patients had an unclassified IBMFS (Table 1). The most common diagnoses among tested patients who had classified IBMFS and unknown mutations was Diamond–Blackfan anemia, followed by Fanconi anemia, Kostmann/severe congenital neutropenia, Shwachman–Diamond syndrome, and dyskeratosis congenita. Among the 120 patients with unclassified IBMFSs the largest group had global bone marrow failure (pancytopenia). Importantly, 20 patients with unclassified IBMFSs had clones/MDS/leukemia at the time they were tested by the NGS gene panel assay.

**Table 1:** Clinical diagnosis of patients tested by the next generation sequencing IBMFS gene panel assay.

Diagnosis	Number of patients tested by the NGS panel
Diamond Blackfan anemia	43
Fanconi anemia	22
Kostmann/Severe congenital neutropenia	16
Shwachman-Diamond syndrome	12
Dyskeratosis congenita	12
Cyclic neutropenia	9
Inherited sideroblastic anemia	7
Congenital dyserythropoietic anemia	6
Congenital amegakaryocytic thrombocytopenia	3
Familial thrombocytopenia	3
Radio-ulnar synostosis	2
Thrombocytopenia absent radii syndrome	2
Reticular dysgenesis	1
Unclassified (total)	120
With pancytopenia	56
With neutropenia	24
With anemia	11
With thrombocytopenia	6
With MDS/leukemia^a^	20
Unknown^b^ (3)	3
Total	258

^a^Some of these patients initially underproductive cytopenia and then developed myelodysplastic syndrome (MDS) and/or leukemia; others presented with clones, myelodysplastic syndrome, or leukemia ^b^unknown

### Pathogenic point variants detected by the NGS gene panel

Pathogenic point variants were revealed in 90 patients (35%) studied by the NGS panel batches 1–3. Among the 138 patients with classified syndromes, pathogenic point variants were identified in 73 patients (53%); whereas among the 120 unclassified patients only 17 (14%) were genotyped. The genes identified to harbor pathogenic point variants by the NGS panel assay are listed in Table 2. The identified genes were related to various disorders on the IBMFS spectrum, including IBMFSs with pancytopenia such as Shwachman–Diamond syndrome, Fanconi anemia, and dyskeratosis congenita, IBMFSs with predominantly anemia, such as Diamond–Blackfan anemia and inherited sideroblastic anemia, IBMFSs with predominantly neutropenia, such as Kostmann/severe congenital neutropenia, and IBMFSs with predominantly thrombocytopenia, such as familial thrombocytopenia. The identified genes function in various cellular pathways, including DNA repair (e.g., *FANCA* and *FANCG*), ribosome biogenesis (e.g., *SBDS* and *RPS19*), telomere maintenance (e.g., *TINF2* and *DKC1*), and hematopoietic signaling (such as *GATA2*). Analysis of point variants from the NGS panel batch 1 and 2 was published previously.^6^ Table 2 shows results of reanalysis of pathogenic point variants from these batches and of those from batch 3.

**Table 2:** Pathogenic point variants detected by the inherited bone marrow failure syndrome NGS gene panel assay.

Gene	OMIM phenotype	Inheri-tance	cDNA alteration	Protein alteration	Zygo-sity	Published or Novel	Conser-vation (GERP)	Sift **	Poli-phen2 **	Mut-Taster **	Mut-Accessor **	Pro-vean **	MAF (gnom-AD)	*N*
**Neutropenia**														
*ELANE* (NM_001972.4)	Cyclic neutropenia,Severe congenital neutropenia	AD	c.182 C > T	p.Ala61Val	hetero	published	3.24	T	D	N	L	N	–	2
			c.466 T > G	p.Trp156Gly	hetero	published*	4.42	D	D	D	H	D	–	1
			c.452 G > C	p.Cys151Ser	hetero	published*	4.42	D	D	D	M	D	–	1
			c.597 + 5 G > A	Splicing site	hetero	published^	n/a	n/a	n/a	n/a	n/a	n/a	–	1
			c.574_581dupGGCCGGCA	p.Val197ArgfsX18	hetero	novel	n/a	n/a	n/a	n/a	n/a	n/a	–	1
			c.176 T > C	p.Leu59Pro	hetero	novel	3.24	D	D	D	H	D	–	1
			c.377 C > T	p.Ser126Leu	hetero	published	2.09	T	B	Da	L	N	–	1
			c.617 T > C	p.Leu206Ser	hetero	novel	4.46	D	D	D	H	D	–	1
			c.212 G > T	p.Cys71Phe	hetero	published	3.24	D	D	D	H	D	–	1
*HAX1* (NM_001018837.1)	Severe congenital neutropenia	AR	c.131 G > A	p.Trp44X	homo	published	n/a	n/a	n/a	n/a	n/a	n/a	–	1
*G6PC3* (NM_138387.2)	Severe congenital neutropenia	AR	c.906dupC	p.Gln305SerfsX82	homo	Published*	n/a	n/a	n/a	n/a	n/a	n/a	–	1
*CXCR4* (NM_003467.2)	Myelokathexis/WHIM Syndrome	AD	c.1000 C > T	p.Arg334X	hetero	published	n/a	n/a	n/a	n/a	n/a	n/a	–	1
*WAS* (NM_000377.2)	Severe congenital neutropenia	XR	c.881 T > C	p.Ile294Thr	hetero	published	4.71	D	D	D	M	D	–	1
**Anemia**														
*RPS19* (NM_001022.3)	Diamond–Blackfan anemia	AD	c.3 G > T	p.Met1Ile	hetero	published	4.53	D	D	D	n/a	D	–	1
			c.10_13delGTTA	p.Val4LeufsX2	hetero	published	n/a	n/a	n/a	n/a	n/a	n/a	–	1
			c.250_251delAG	p.Arg84LysfsX69	hetero	published	n/a	n/a	n/a	n/a	n/a	n/a	–	1
			c.185 G > A	p.Arg62Gln	hetero	published	4.3	D	B	D	H	D	–	4
			c.16delG	p.Val6X	hetero	published*	n/a	n/a	n/a	n/a	n/a	n/a	–	1
			c.155 G > A	p.Trp52X	hetero	published	4.57	n/a	n/a	n/a	n/a	n/a	–	1
			c.3 G > A	p.Met1Ile	hetero	published	4.53	D	D	D	n/a	D	–	1
*RPL11* (NM_000975.5)	Diamond–Blackfan anemia	AD	c.60_61delCT	p.Cys21SerfsX33	hetero	published	n/a	n/a	n/a	n/a	n/a	n/a	–	2
			c.158-1 G > C	Splicing site	hetero	novel^	n/a	n/a	n/a	n/a	n/a	n/a	–	1
			c.372 C > G	p.Ile124Met	hetero	published*	5.94	D	P	D	H	D	–	1
*RPL5* (NM_000969.5)	Diamond–Blackfan anemia	AD	c.83delC	p.Thr28MetfsX10	hetero	novel	n/a	n/a	n/a	n/a	n/a	n/a	–	1
			c.174_175 delAG	p.Arg58ArgfsX53	hetero	published*	n/a	n/a	n/a	n/a	n/a	n/a	–	1
			c.166_169delACAA	p.Asn57fsX12	hetero	published	n/a	n/a	n/a	n/a	n/a	n/a	–	1
*RPL35A* (NM_000996.2)	Diamond–Blackfan anemia	AD	c.78_80delCTT	p.Leu28del	hetero	published	n/a	n/a	n/a	n/a	n/a	n/a	–	1
*RPS24* (NM_033022.3)	Diamond–Blackfan anemia	AD	c.4-2 A > G	Splicing site	hetero	published*^	n/a	n/a	n/a	n/a	n/a	n/a	–	1
			c.1 A > G	p.Met1?	hetero	published	5.17	D	P	D	n/a	N	–	1
*RPS26* (NM_ 001029.5)	Diamond–Blackfan anemia	AD	c.4-32_21del (12)	Splicing site (branch point)	hetero	published*	n/a	n/a	n/a	n/a	n/a	n/a	–	1
			c.243delC	p.Ser81ArgfsX3	hetero	published*	n/a	n/a	n/a	n/a	n/a	n/a	–	1
			c.1 A > G	p.Met1?	hetero	published	5.92	D	P	D	n/a	D	–	1
			c.77 G > C	p.Cys26Ser	hetero	novel	4.43	D	P	D	M	D	–	1
*RPS29* (NM_001032.4)	Diamond–Blackfan anemia	AD	c.63-3 C > A	Splicing site	hetero	published*^	n/a	n/a	n/a	n/a	n/a	n/a	–	1
*RPS7* (NM_001011.4)	Diamond–Blackfan anemia	AD	c.398 T > C	p.Leu133Ser	hetero	published*	4.6	D	D	D	M	D	–	1
*RPS10* (NM_001014.4)	Diamond–Blackfan anemia	AD	c.337 C > T	p.Arg113X	hetero	published	n/a	n/a	n/a	n/a	n/a	n/a	–	1
			c.457-3 C > G	Splicing site	hetero	novel^	n/a	n/a	n/a	n/a	n/a	n/a	–	1
			c.239 G > A	p.Arg80His	hetero	novel	5.19	D	P	D	H	D	–	1
*SLC25A38* (NM_017875.4)	Sideroblastic anemia	AR	c.560 G > A	p.Arg187Gln	homo	novel	3.98	D	D	D	M	D	3.978E−06	3
*CDAN1* (NM_13877.4)	Congenital dyserythropoietic anemia I	AR	c.2081 C > T/c.2015C > T	p.Pro694Leu/p.Pro672Leu	comp hetero^#^	novel/published	5.77	D	D	D	M	D	0.000008017/0.00008498	2
							5.77	T	D	D	M	D		
*SEC23B* (NM_001172745.2)	Congenital dyserythropoietic anemia II	AR	c.1648C > T/c.211 A > C	p.Arg550X	comp hetero^#^	novel/novel	3.53	n/a	n/a	n/a	n/a	n/a	0.0000541/–	1
				p.Asn71His			5.29	D	D	D	H	D		
**Thrombocytopenia**														
*RBM8A* (NM_005105.4)	Thrombocytopenia-absent radius syndrome	AR	c. -21G > A	5’UTR variant	homo	published	n/a	n/a	n/a	n/a	n/a	n/a	0.01794	2
*ANKRD26* (NM_014915.2)	Thrombocytopenia	AD	c.4976dupA	p.Ile1659TyrfsX3	hetero	published*	n/a	n/a	n/a	n/a	n/a	n/a	8.048E−06	1
*MYH9* (NM_002473.5)	Macro thrombocytopenia	AD	c.3493 C > T/c.4562 A > G	p.Arg1165Cys	hetero	published	4.92	D	D	D	H	D	–	1
				p.His1521Arg	hetero	novel	5.25	D	D	D	H	D	–	1
*MPL* (NM_005373.2)	Congenital amegakaryocytic thrombocytopenia,	AR	c.304 C > T	p.Arg102Cys	homo	novel	5.56	D	D	D	M	D	1.193E−05	1
*WAS* (NM_000377.2)	Wiskott–Aldrich syndrome	XR	c.157_162delCTGTAC	p.Leu53_Tyr54del	hemi	novel	n/a	n/a	n/a	n/a	n/a	n/a	–	1
*MASTL* (NM_001172303.2)	Thrombocytopenia	AD	c.811 + 2 T > G	Splicing site	hetero	published	n/a	n/a	n/a	n/a	n/a	n/a	7.965E−06	1
**Pancytopenia**														
*FANCA* (NM_000135.2)	Fanconi anemia	AR	c.1115_1118delTTGG	p.Val372AlafsX42	homo	published	n/a	n/a	n/a	n/a	n/a	n/a	6.717E−05	2
			c.2830_2831InsCAGCTTCAGGTTGAATTTC	p.Asp944GlufsX12	homo	published*	n/a	n/a	n/a	n/a	n/a	n/a	–	1
			c.3788_3790delTCT	p.Phe1263del	homo	published	n/a	n/a	n/a	n/a	n/a	n/a	9.929E−05	1
			c.1645C > T	p.Gln549X	homo	published	n/a	n/a	n/a	n/a	n/a	n/a	–	1
			c.2851 C > T/	p.Arg951Trp/	comp hetero^#^	published/published	2.41	D	D	D	n/a	D	0.00002386/–	1
			c.1470 G > A	p.Gln490Gln			n/a	n/a	n/a	n/a	n/a	n/a		
*FANCJ/BRIP1* (NM_032043.2)	Fanconi anemia	AR	c.2392 C > T	p.Arg798X	homo	published	n/a	n/a	n/a	n/a	n/a	n/a	0.0001581	2
*FANCG* (NM_004629.1)	Fanconi anemia	AR	c.1480 + 1 G > C	Splicing site	homo	published	n/a	n/a	n/a	n/a	n/a	n/a	1.988E−05	1
*FANCE* (NM_021922.2)	Fanconi anemia	AR	c.1111 C > T	p.Arg371Trp	homo	published	3.48	D	D	D	M	D	0.0001193	1
*ERCC4/FANCQ* (NM_005236.2)	Fanconi anemia	AR	c.2065 C > A	p.Arg689Ser	homo	published	5.58	D	D	D	H	D	7.954E−06	1
*FANCI* (NM_001113378.1)	Fanconi anemia	AR	c.2776_2789delinsG	p.Val926AlafsX38	homo	novel	n/a	n/a	n/a	n/a	n/a	n/a	0.0002704	1
*FANCP/ SLX4* (NM_032444.4)	Fanconi anemia	AR	c.1366 G > A	p.Glu456Lys	homo	novel	4.95	n/a	n/a	Da	n/a	n/a	–	1
*TINF2* (NM_001099274.3)	Dyskeratosis congenita	AD	c.844 C > G	p.Arg282Gly	hetero	published*	5.16	D	D	D	M	N	–	1
			c.845 G > A	p.Arg282His	hetero	published	5.16	D	D	D	M	N	–	1
			c.734 C > A	p.Ser245Tyr	hetero	published	0.987	D	B	N	N	N	0.001009	1
*DKC1* (NM_001142463.2)	Dyskeratosis congenita	XR	c.112delA/ c.116InsC	p.Ile38Ser+	hemi	novel/	n/a	n/a	n/a	n/a	n/a	n/a	–	1
			c.949 C > T	p.Lys39Thr		novel	n/a	n/a	n/a	n/a	n/a	n/a	–	
				p.Leu317Phe	Hemi	published	5.85	D	P	D	H	D	–	1
*RTEL1* (NM_032957.4)	Dyskeratosis congenita/severe aplastic anemia	AD or AR	c.1373 C > T/	p.Thr458Met/	comp hetero	published*/	-0.805	D	B	N	L	N	0.0004542/0.00005245	1
			c.1416 G > C	p.Lys472Asn		published	0.204	D	B	D	M	D		
			c.3442delC/	p.Gln1149Argfs	comp hetero^#^	published*/	n/a	n/a	n/a	n/a	n/a	n/a	–/–	1
			c.49 C > T	X96/p.Pro17Ser		published*	4.86	D	D	D	M	D		
*TERC* (NR_001566.1)	Dyskeratosis congenita	AD Or AR	n.37 A > G/n.216_229del	Not applicable	Comp hetero	Published/	n/a	n/a	n/a	n/a	n/a	n/a	2.669E−05	1
			GGCGGGTCGCCTGC	Not applicable		published	n/a	n/a	n/a	n/a	n/a	n/a	–	
			n.83 T > C	Not applicable	hetero	published*	n/a	n/a	n/a	n/a	n/a	n/a	–	1
*TERT* (NM_198253.2)	Dyskeratosis congenita	AD or AR	c. 2014C > T	p.Arg672Cys	hetero	novel	3.76	D	B	N	M	D	0.0002318	2
			c.1234 C > T	p.His412 Tyr	hetero	published^$^	2.32	T	B	N	L	N	0.003248	1
*SBDS* (NM_016038.2)	Shwachman-Diamond syndrome	AR	c.258 + 2 T > C	Splicing site	homo	published	n/a	n/a	n/a	n/a	n/a	n/a	0.003879	1
			c.120delG/c.258 + 2T > C	p.Ser41AlafsX18/splicing site	comp hetero^#^	published*/published	n/a	n/a	n/a	n/a	n/a	n/a	0.0000079/6/0.003879	2
							n/a	n/a	n/a	n/a	n/a	n/a		
			c.127 G > T/c.258 + 2 T > C	p.Val43Leu/splicing site	comp hetero^#^	published*/published	3.31	D	P	D	M	N	0.001144/0.003879	1
							n/a	n/a	n/a	n/a	n/a	n/a		
			c.183_184delTAinsCT/c.258 + 2 T > C	p.Lys62X/splicing site	comp hetero	published/	n/a	n/a	n/a	n/a	n/a	n/a	−/0.003879	2
						published	n/a	n/a	n/a	n/a	n/a	n/a		
**Myelodysplastic syndrome**														
*GATA2*(NM_001145661.1)	Emberger syndrome,	AD	c.1009 C > T	p.Arg337X	hetero	published	3.33	n/a	n/a	n/a	n/a	n/a	–	1
*SOS2* (NM_006939.4)	Noonan syndrome	AD	c.1868G > A	p.Arg623His	hetero	published	4.53	D	B	D	M	D	8.591E−05	1

*AD* autosomal dominant, *AR* autosomal recessive, *hemi* hemizygous, *hetero* heterozygous, *homo* homozygous, *LP* likely pathogenic, *N* number of patients, *VUS* variant of unknown/uncertain significance

^#^Indicated presumed zygosity. Compound heterozygosity was determined based on the observation of two mutations on different alleles by the BAM file data. Otherwise the zygosity of patients with a disease whose associated gene harbored two mutations were considered as having “presumed compound heterozygosity”

*Published previously as novel by our group (Ghemlas et al, J. Med. Genet. 2015).

**SIFT: D, damaging; T, tolerated

**POLYPHEN2: D, probably damaging; P, possibly damaging; B, benign; N, neutral **PROVEAN: D, damaging; N, neutral

**MutationTaster: disease causing automatic (Da); Disase causing (D); polymorphisam (SNP), polymorphism automatic (SNPa)

**MutattionAssessor: H, high; M, medium; N, neutral; L, low

^$^Published in the Telomerase database (http://telomerase.asu.edu/)

^Splicing site variant was analyzed by the Human Splicing Finder and found to be pathogenic

The cases described in this table were analyzed in three batches. Analysis of point variants from batch 1 and 2 was published previously (Ghemlas et al., Journal of Medical Genetics 2015; 52; 575–84). This table shows results of re-analysis of pathogenic point variants from these batches and of those from batch 3. There are some overlap between data in this table and data in supplemental tables 2–5 of the publication in the Journal of Medical Genetics

### Overall detection of CNVs by the NGS panel

**Fig. 1:**
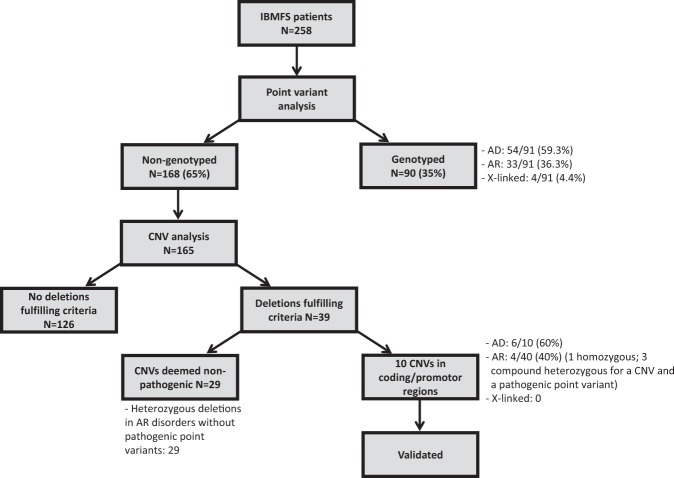
Workflow of cohort analysis and overall rates of pathogenic variant detection.

Of the 258 patients analyzed by the panel 168 were not found to have pathogenic point variants by the panel. Samples from 165 of the patients without identified pathogenic point variants were subjected to CNVs analysis using a computerized software program. Of the 165 patients, 10 (6%) were found to have pathogenic deletions in IBMFS genes (Fig. 1) using the strategy described in the Methods. All identified deletions were validated. Detailed description of the deletion and subjects are presented in the following paragraphs and in Table 3.

**Table 3:** Pathogenic copy number variants identified by the inherited bone marrow failure syndrome NGS gene panel.

Patient no.	Diagnosis at the time of testing	Hematological abnormalities	Main physical malformations	Time from diagnosis to CNV detection (months)	Gene (RefSeq accession)	Zygosity	Deletion region	Ratio	Dispersion	Number of reports in patients^a^	Number of reports in helathy controls^a^
1	Diamond–Blackfan anemia	Severe anemia, decreased bone marrow erythopiesis. normal eADA good respond to corticosteroids	Scoliosis	411	*RPS19* (NM_001022)	hetero	Promoter –Exon 5	0.32–0.38	0.0016-0.0039	1	0
2	Diamond–Blackfan anemia	Severe anemia, decreased bone marrow erythopiesis, normal eADA, partial response to corticosteroids	Large almond-shaped eyes	120	*RPS19* (NM_001022)	hetero	Promoter –Exon 5	0.28–0.40	0.0012-0.0088	1	0
3	Diamond–Blackfan anemia	Severe anemia, decreased bone marrow erythopiesis, normal eADA¸no response to corticosteroid, underwent HSCT	None	67	*RPS19* (NM_001022)	hetero	Promoter –Exon 5	0.27–0.33	0.002-0.019	1	0
4	Diamond–Blackfan anemia	Severe anemia, decreased bone marrow erythopiesis, elevated eADA, no response to corticosteroid, received chronic red cell transfusion	Short stature, atrial septal defect, sensorineural hearing loss, developmental delay	354	*RPL5* (NM_000969)	hetero	Promoter–Exon7	0.20–0.35	0.02-0.26	5	1 (intronic)
5	Diamond–Blackfan anemia	Severe anemia, decreased bone marrow erythopiesis, elevated eADA, high HbF, good response to corticosteroid	Failure to thrive, bilateral cleft lip & palate, thumb abnormality, tracheomalacia, developmental delay	161	*RPL11* (NM_000975)	hetero	Promoter –Exon6	0.30–0.34	0.005-0.0096	5	1 (intronic)
6	Thrombocytopenia absent radii	Thrombocytopenia, transient need for platelet transfusion in infancy	Absent radii	273	*RBM8A* (NM_005105)	comp hetero with c.-21G > A	Promoter –Exon6	0.31–0.34	0.001-0.01	155	3 (hetero)
7	Thrombocytopenia absent radii	Thrombocytopenia, transient need for platelet transfusion in infancy	Absent radii	79	*RBM8A* (NM_005105)	comp hetero with c.-21G > A	Promoter –Exon6	0.31–0.35	0.015-0.01	155	3 (hetero)
8	Myelodysplastic syndrome (CNV led to diagnosis of GATA2-related disorder)	Moderate pancytopenia, hypocellular bone marrow, dyserythopoiesis, marrow trisomy 8, no available data available about treatment.	Retrognathia, cutaneous warts, developmental delay, attention-deficit/hyperactivity disorder	239	*GATA2* (NM_032638)	hetero	Promoter –Exon5	0.30–0.39	0.002-0.008	16	0
9	Unclassified pancytopenia (CNV led to diagnosis of Fanconi anemia)	Severe thrombocytopenia, mild neutropenia, hypocellular bone marrow, dyserythropoiesis, reduced megakaryocytes, no response to immunosuppressive therapy, underwent HSCT	Low birth weight, short staure, café-au-lait spots, absent one kidney, developmental delay	133	*FANCA (NM_000135)*	homo	Promoter –Exon5	0.002–0.004	0.011-0.067	16	7 (hetero)
10	Unclassified pancytopenia	Thrombocytopenia, later on pancytopenia, markedly hypocellular bone marrow, partial long-term response to danazol	Short stature, failure to thrive, microcephaly, global developmental delay, seizures, brain demyelination, brittle hair, mid face hypoplasia, bulging eyes, low set ears, kyphosis, scoliosis, right double joint arms	128	*PARN* (NM_002582)	comp heterowith c.1045 C > T	Exon15– Exon18	0.32–0.39	0.003-0.03	18	1 (hetero)

*CNV* copy number variation, *comp hetero* compound heterozygosity, *hetero* heterozygosity, *eADA* erythrocyte adenosine deaminase, *HSCT* hematopoietic stem cell transplantation

^a^Data about reported patients and controls were taken from the DECIPHER, ClinVAR, DGV, and gnomAD databases. Reported cases might have reported in more than one database

### Detection of CNVs in patients with previously established clinical diagnosis

**Fig. 2:**
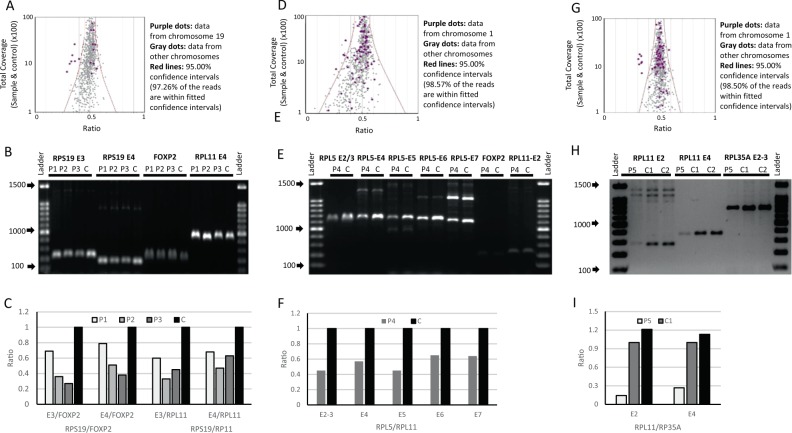
Deletions in patients with Diamond Blackfan anemia. **a** Representative raw data dispersion with 95% confidence intervals from one of the three patients with *RPS19* deletions (Patient 2). The dispersion analysis showed that 97.26% of the reads were within fitted interval. The purple dots represent data from chromosome 19 and show clear separation of dots outside the whole data (the *RPS19* deletion) from other dots on chromosome 19 (also purple) data and on other chromosomes (gray dots). **b** Gel electrophoresis shows clear reduction in the quantity of signal of *RPS19* exon 3 and exon 4 in the patients compared to controls. In this experiment *FOXP2* and *RPL11* were used as internal controls. **c** Results of band densitometry from the gel electrophoresis. **d** Raw data dispersion with 95% confidence intervals from patient 4 are shown. Totally, 98.57% of the read were within fitted interval. The purple dots on the left show clear separation of the *RPL5* deletion from the whole data and from the chromosome 1 data in each of the patient samples. **e** Gel electrophoresis shows clear reduction in the quantity of signal of *RPL5* exon 2–7 in the patient compared to controls. In this experiment *FOXP2 and RPL11* were used as internal controls. **f** The lower panel shows the results of band densitometry from the gel electrophoresis. **g** An *RPL11* gene deletion in Patient 5 is shown. The figure shows the raw data dispersion with 95% confidence intervals from patient 5. Totally, 98.50 % of the reads were within fitted interval. The purple dots on the left shows clear separation of the *RPL11* deletion from the whole data and from the chromosome 1 data in each of the patient samples. **h** Gel electrophoresis shows clear reduction in the quantity of signal of *RPL11* exon 2 and 4 in the patient compared to controls. In this experiment *RPL35A* exon 7 was used as an internal control. **i** Band densitometry from the gel electrophoresis is shown. E indicates exon, P indicates a patient number, and C indicates a healthy control subject.

The largest group of patients with identified deletions were those with Diamond–Blackfan anemia and included five patients. Three of these patients had a heterozygous deletion that encompassed the entire *RPS19* gene (Fig. 2a–c and Supplementary Table 3). Despite being siblings, having severe anemia since infancy, markedly reduced bone marrow erythropoiesis and normal erythrocyte adenosine deaminase (eADA) activity levels, these patients had otherwise variable clinical phenotypes and outcome. Patient 1 had scoliosis, patient 2 had large almond-shaped eyes, and patient 3 had no physical malformations. These patients were treated with prolonged courses of oral corticosteroids with varied responses. Patient 1 responded well to corticosteroid treatment. Patient 2 had only a partial response; his hemoglobin was still lower than the normal level for age and he required few red blood cell transfusions when he had intercurrent infections. Patient 3 did not respond to corticosteroids and underwent matched unrelated hematopoietic stem cell transplantation (HSCT) but developed graft failure and died from sepsis after a second HSCT.

Patient 4 had one copy deletion in *RPL5* from the promoter region to exon 7 (Fig. 2d–f and Supplementary Table 4). In addition to severe anemia, she had short stature, a large atrial septal defect, sensorineural hearing loss and developmental delay. The patient did not respond to corticosteroid therapy and required chronic red blood cell transfusions.

One copy deletion of the entire *RPL11* gene was identified in Patient 5 (Fig. 2g–i and Supplementary Table 5). This patient presented with severe macrocytic anemia, reticulocytopenia and high hemoglobin F at 2 years of age. Bone marrow examination demonstrated pure red cell aplasia. Activity of eADA was elevated. He was born with low birth weight and also suffered from failure to thrive, bilateral complete cleft palate and cleft lip, tracheomalacia, subglottic stenosis, thumb abnormality and developmental delay. He had a good response to corticosteroid therapy.

Patients 6 and 7 had a clinical diagnosis of thrombocytopenia with absent radii syndrome. Using the NGS panel assay we identified compound heterozygosity for a deletion on one allele of *RBM8A* and a single pathogenic point variant on the other allele. The cases have been previously reported,^6^ and the deletions were also detected by Affymetrix SNP array.

### Establishment of a syndromic diagnosis in patients with unclassified IBMFSs based on CNV data

**Fig. 3:**
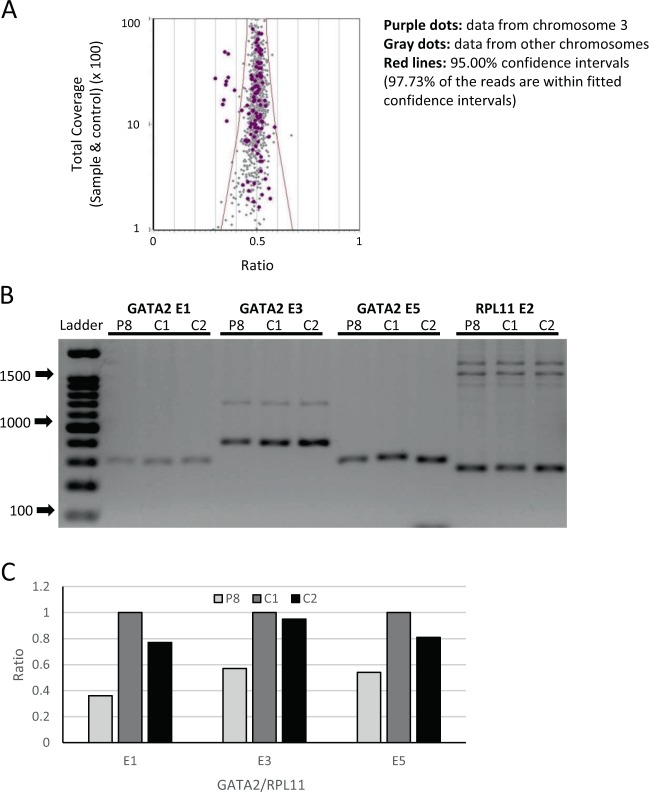
Deletion of *GATA2* gene in a patient with myelodysplastic syndrome. **a** The figure shows the raw data dispersion with 95% confidence intervals from patient 8. 97.73% of the reads were within fitted interval. The purple dots on the left shows clear separation of the *GATA2* deletion from the whole data and from the chromosome 3 data in each of the patient samples. **b** Gel electrophoresis shows clear reduction in the quantity of signal of *GATA2* exon 1, 3, and 5 in the patient compared to controls. In this experiment *RPL11* exon 2 was used as internal controls. **c** The panel shows the results of band densitometry from the gel electrophoresis.

In three cases with an unclassified IBMFS, detection of CNVs led to establishment of a syndromic diagnosis. Patient 8 had MDS. He was found to carry a germline deletion of the entire *GATA2* gene (Fig. 3a–c and Supplementary Table 6). He had moderate pancytopenia, high mean corpuscular volume, developmental delay, attention-deficit/hyperactivity disorder, retrognathia and cutaneous warts on his hands. Bone marrow revealed hypocellular specimen, decreased trilineage hematopoiesis and dyserythropoiesis. Bone marrow cytogenetic analysis showed 46,XY,+1,der(1;7)(q10;p10)[23]/47,idem,+8[5]/46,XY[23].

**Fig. 4:**
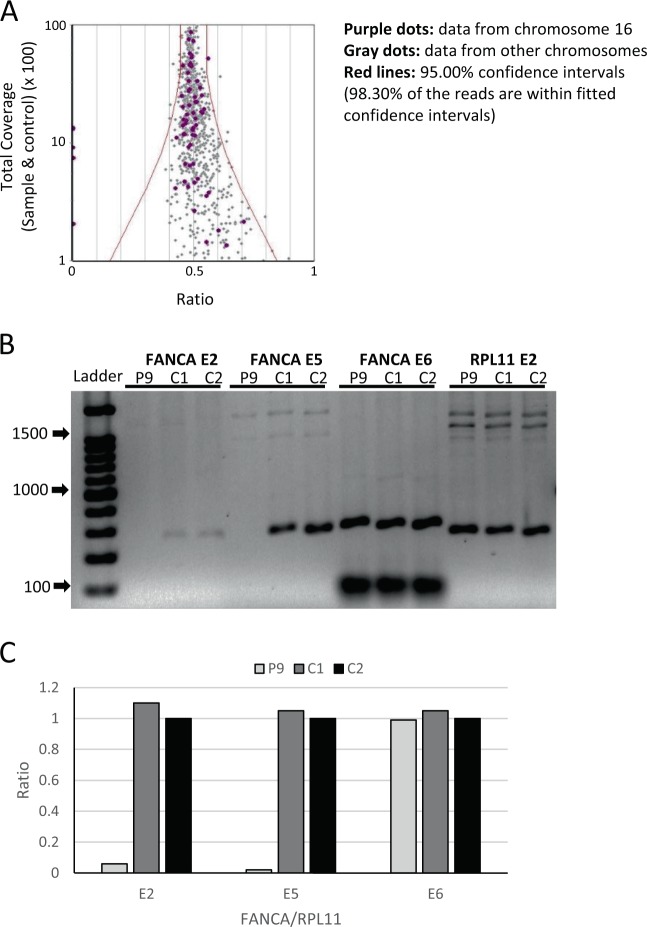
Deletion of *FANCA* gene in a patient with unclassified inherited bone marrow failure syndrome. **a** The raw data dispersion with 95% confidence intervals from patient 9 are shown. Totally, 98.30% of the read were within fitted interval. The purple dots on the left exhibit ratio of <0.05 and are clearly separated from the whole data and from the other reads of chromosome 16. **b** Gel electrophoresis shows the absence of amplified *FANCA* exon 2 and 5 in the patient compared to controls. In this experiment, FANCA exon 6 and *RPL11* exon 2 were used as internal controls. **c** The panel shows the results of band densitometry from the gel electrophoresis.

Patient 9 had an unclassified IBMFS with pancytopenia and was found to have a homozygous deletion of the *FANCA* gene from exon 1 to exon 5 (Fig. 4a–c and Supplementary Table 7). The patient presented with severe thrombocytopenia and mild neutropenia at 8 years of age. Evaluation of his disease revealed low birth weight, short stature, café-au-lait spots, absent right kidney, and developmental delay. This patient did not respond to immunosuppressive therapy for aplastic anemia and eventually died after HSCT. No data about chromosome fragility testing and genetic testing were reported to the registry.

Patient 10 presented with thrombocytopenia at 1 year 6 months of age and then progressed to pancytopenia. This patient had multiple anomalies as described in Table 3 and in our previous published article.^21^ The patient was initially diagnosed with an unclassified IBMFS. Due to an identified presence of compound heterozygosity in *PARN* and further functional assays, the patient was classified as having dyskeratosis congenita.^21^ Analysis of the NGS reads by NextGene revealed both the heterozygous deletion and pathogenic point variant. Compound heterozygosity was confirmed by parental testing.^21^

## Discussion

CNVs are an important cause of many genetic disorders including IBMFSs.^22^ However, methods to detect CNVs lack sensitivity, and frequently multiple diagnostic approaches and tests are required to detect them. Herein, we investigated the ability to uncover CNVs from a method primarily designed for nucleotide-level analysis. We identified CNVs in a proportion of the cases where pathogenic point variants were not found. Currently, NGS gene panels are the standard method to search for genetic mutations. Ideally, these panels would also used to detect CNVs at the same time. This strategy would save time and decrease overall cost of investigation. In the case of IBMFSs, the mutations (large chromosomal changes, smaller CNVs, indels, and other point mutations) and location (protein and nonprotein encoding genes) are variable.^6,10^ Consequently, the time and cost of genetic testing, if not done efficiently, could be prohibitive. This is particularly important since many IBMFSs patients need treatment urgently. Hence, extracting the maximum amount of information out of each test is critically important. Our study indicates that using NGS gene panel assay for both point mutations and detection of CNVs increases the information that can be retrieved from the assay.

Results of our study demonstrate that analysis of NGS gene panel assays for CNVs can not only establish a genetic diagnosis (such as identifying *RPL5* mutations in patients with DBA), but can also identify the specific diagnosis in unclassified or idiopathic bone marrow failure syndromes. In this study, a diagnosis of Fanconi anemia was made in a patient with unclassified IBMFSs and a diagnosis of *GATA2*-related disorder in another with idiopathic MDS. The identification of a genetic diagnosis in the case of DBA for example is critically important for genetic and family counseling. Similarly, establishing a specific diagnosis of Fanconi anemia in a patient with aplastic anemia is extremently important given the treatment for Fanconi anemia is completely different from that of idiopathic aplastic anemia. Importantly, although positive chromosome breakage testing is the hallmark of Fanconi anemia, in rare cases it is negative or difficult to interpret due to somatic genetic correction and lymphocyte mosaicism in the peripheral blood chromosome fragility testing. Also, establishing a diagnosis of *GATA2*-related disorder in a patient with MDS would completely change the approach to counseling, screening and selection of family members as donors for bone marrow transplantation for the affected subject.

To our knowledge, this is the first study to analyze an IBMFS gene panel by computerized software to detect small and large size CNV. This software uses normalized coverage value to compare with a defined number of controls, which we set at 10. Most of the detected CNVs were successive exons where at least one of them showed a normalized coverage ratio of ≤0.33 in the case of monoalleic deletion and a normalized coverage ratio of ≤0.05 in the case of bialleic deletion. Validation by an additional method is recommended in case of borderline normalized ratios.

Detection of CNVs from NGS reads using the NextGene software has some limitations. Even positive tests included the combination of low dispersion and normalized coverage ratios that were found in 3 sets of control data but some borderline results might still be positive and may require validation. The combination of developing new algorithms to interpret borderline results and correlating data with clinical phenotype may improve categorization of results as true positive versus false positive. CNV duplications are difficult to identify by the software. Further, some duplicated fragments may not reside in the gene region and may not disrupt the coding sequence.

In summary, NGS gene panels can be used to detect CNVs by careful analysis of normalized coverage values. In our analysis small CNVs encompassing one to few exons were detected from an IBMFS NGS gene panel assay. Due to time and cost advantages, we recommend that NGS gene panels routinely analyze for CNVs before moving on to additional mutation detection strategies such as CNV microarray or whole exome/genome sequencing. Recently the cost of a clinical whole-exome sequencing (WES) test dropped substantially (about $3500 (USA)) and became closer to clinical NGS panels (about $1500 (USA)). Therefore, performing WES as a first line genomic diagnostic test can be considered, particularly for designing virtual panels and only analyzing genes of interest. In such cases, similar strategies to those described in the present study can be applied to obtain CNV data from WES. The results of our work have a significant clinical relevance not only to the field of bone marrow failure, MDS and leukemia, but also to many other disciplines where genetic testing by NGS gene panels became the standard of care. Our study also provides additional tools for research on the prevalence of CNVs in various genetic disorders and on CNV detection by whole exome/genome sequencing.

## Methods

### Selection of patients

The patients included in this study were prospectively enrolled in the Canadian Inherited Marrow Failure Registry (CIMFR) and did not have known disease-causing mutations. The CIMFR and the experiments outlines in this manuscripts were approved by the Research Ethics Boards of the Hospital for Sick Children and the participating tertiary medical centers in Canada. The study is conducted in accordance with the Declaration of Helsinki, and informed written consent was obtained from all patient or their guardian prior to enrollment. Patients who fulfilled diagnostic criteria for an IBMFS^23^ in any of the 17 participating centers in Canada were enrolled in the CIMFR since 2001, by the site research team. The vast majority of children with IBMFSs are treated in one of the participating CIMFR site centers. Patients information was collected at study entry and periodically thereafter.

The eligibility criteria for the CIMFR included evidence of chronic bone marrow failure in addition to either a family history of an IBMFS, or physical malformations, or presentation earlier than one year of age. Patients enrolled in the CIMFR who had positive genetic testing for an IBMFS gene were excluded from this analysis. When possible, each case was assigned a specific syndromic diagnosis by the participating center. Diagnoses were reviewed centrally, and if necessary adjusted based on published diagnostic criteria of specific IBMFSs^1–3^ after verification with the respective center. Cases that fulfilled the eligibility criteria, but did not meet the clinical, laboratory and genetic diagnostic criteria for any known IBMFS subtype were defined as unclassified IBMFSs.^24^

### NGS panel assay

Genomic DNA was extracted from peripheral blood, bone marrow fibroblasts, skin fibroblasts or expanded peripheral blood T-cells. Comprehensive NGS panel of known IBMFS genes was designed. Overall, 72, 77 and 141 genes, were included in the first, second, and third analyzed batch of patients, respectively (Supplementary Table 1). The Haloplex Capture Kit (Agilent Technologies, Santa Clara, CA) was used for DNA library preparation according to the manufacturer instructions. Briefly, the assay was based on a hybridization oligonucleotide pool, which covers coding regions, 50 bp flanking intronic regions that included splicing sites, 3′-untranslated regions that included potential translation regulatory elements, and upstream promoter regions. The oligonucleotides were 150 mers with 3× tiling and a maximum of 10 bp overlap between oligonucleotides. The panel design was submitted to the Agilent HaloPlex Design Wizard program (http://www.halogenomics.com/haloplex/custom-reagent-kits). Targeted fragments were amplified and were sequenced on the Illumina HiSeq2500 platform as previous described.^6^

### Variant analysis and filtering strategy

The algorithm used to filter non-relevant point variants and the software programs and websites used to predict protein damage, conservation and minor allele frequency of pathogenic point variants are as previously described.^6^ Briefly, variants were defined as “pathogenic” if they had been reported as disease-causing in public databases. Novel variants were considered “most likely pathogenic” if (1) they appeared in allelic dosage that was consistent with the known inheritance mode of the disease, (2) evolutionary conserved amino acid/s are affected, (3) the minor allele frequency was <0.001 (4) the variant was considered damaging by at least half of the following prediction software programs: PolyPhen2, SIFT, MutationTaster, MutationAssessor, Provean. Splicing variants were assessed by the Human Splicing Finder software program. Variants that were reported in databases as having unknown significance (usually due to only one or two reported cases), but fulfilled the above criteria were considered as “most likely pathogenic”. Variants that fulfilled most but not all the above criteria remained of unknown significance.

### CNV analysis by normalized coverage values

Patients who were found to have no pathogenic point variants by the NGS gene panel assay were analyzed for CNVs. We used the NextGene software program, CNV Tools, to compare the coverage ratio of specific regions in a test sample to ten samples of control projects, which were samples of sex-matched healthy control subjects or patients with other disorders that are not expected to have mutations in the analyzed gene. The beta-binomial model was used to evaluate dispersion. The Hidden Markov Model was used to calculate normalized count ratio and make a classification of specific regions as CNV. To minimize false positive results, we selected calls with dispersion of ≤0.01, minimal normalized read counts of 100, minimal region length of 50 bp and percentage of regions in which CNV calls are expected to be made is ≤5%. After the test sample and 10 control sex-matched samples were loaded, a comparison was made first to the average coverage of all 10 controls; second, to median coverage of all 10 controls; third, to the coverage of one control subject, whose coverage was closest to the test sample. Only regions that were deemed deleted in all three comparisons, were considered true deletions. Short variant calls (<50 bp) were excluded since most often they represent random background noise.

Candidate heterozygous deletions were considered and selected for validation studies if they fulfilled the following criteria: (1) the raw data dispersion was ≥95% and the normalized coverage ratio was ≤0.33 in all three types of comparisons to controls as indicated above; (2) multiple successive exons with raw data dispersion of >95% and at least one of the exons shows normalized coverage ratio of ≤0.33 in all three types of comparisons to controls as indicated above.

Results were considered candidate homozygous deletions and were selected for validation if they fulfilled the following criteria: (1) the raw data dispersion was >95% and the normalized coverage ratio was <0.05 in all three types of comparisons to controls as indicated above; (2) the raw data dispersion was >95% and multiple successive exons that at least one of them shows normalized coverage ratio of <0.05 in all three types of comparisons to controls as indicated above. Determination of CNV frequency in the general population and degree of overlap with previously reported CNVs was done automatically by the software using the Database of Genomic Variants,^25^ and manually using the following databases: Human Gene Mutation Database (http://www.hgmd.cf.ac.uk/), ClinVar (https://www.ncbi.nlm.nih.gov/clinvar/), DatabasE of Chromosomal Imbalance and Phenotype in Humans using Ensembl Resources (https://decipher.sanger.ac.uk/) and Genome Aggregation Database (gnomAD, https://gnomad.broadinstitute.org/).

### Polymerase chain reaction

DNA was amplified using customized primers flanking the regions that was found to be deleted by the NGS panel. The primer sets are described in Supplementary Table 2. Amplified DNA fragments were separated by agarose gel electrophoresis and were visualized by ultraviolet light. Band densitometry was determined using the ImageJ software.

### Affymetrix SNP array 6.0

DNA was processed, hybridized to Affymetrix Genome-Wide Human SNP array 6.0 (Affymetrix Inc., Santa Clara, CA, USA) and scanned as previously described.^21^ Genotyping calls were determined using the Birdseed v.2 algorithm as described.^6^ CNV were considered novel if they did not appear in healthy controls from The Center of Applied Genomics (Hospital for Sick Children, Toronto, Canada).

### Reporting summary

Further information on research design is available in the Nature Research Reporting Summary linked to this article.

## Supplementary information

Supplemental Material

Reporting Summary Checklist

## Data Availability

The data that support the findings of this study are available on request from the corresponding author [Y.D.]. The data are not publicly available since this can compromise research participant consent.
