# Supplementary material for: Reanalysing genomic data by normalized coverage values uncovers CNVs in bone marrow failure gene panels
Source: NPJ Genom Med. 2019 Dec 9;4:30. doi: 10.1038/s41525-019-0104-9 (PMC6901453; doi:10.1038/s41525-019-0104-9)

Supplementary Data

Re-analysis of next generation sequencing gene panel data by normalized coverage values unravel CNVs in bone marrow failure syndromes

Supanun Lauhasurayotin, Cuvelier GDE, Robert J Klaassen, Conrad Fernandez, Yves D. Pastore, Sharon Abish, Reena Pabari, MacGregor Steele, Lawrence Jardine, Vicky R Breakey, Josee Brossard, Roona Sinha, Mariana Silva, Lisa Goodyear, Jeffrey H. Lipton, Bruno Michon, Mark J. Belletrutti, Lillian Sung, Scherer W Stephen, Iren Shabanova, Hongbing Li, Bozana Zlateska, Santhosh Dhanraj, Michaela Cada, Yigal Dror.

Supplementary Table 1: Genes included in the next generation sequencing gene panel assay in the first, second and third batch

NGS Panel Assay Batch 1	NGS Panel Assay Batch 2	NGS Panel Assay Batch 3
ABCB7, AK2, ALAS2, ANKRD26, BRCA2, BTHS, CDAN1, CTC1, CXCR4, DKC1, ELANE, FAAP95, FANCA, FANCC, FANCD2, FANCE, FANCF, FANCG, FANCI, FANCJ, FANCL, FANCM, FANCP, FECH, G6PC3, GATA1, GATA2, GFI1, GLRX5, GP1BA, HAX1, HOXA11, KLF1, LIG4, MASTL, MPL, MYH9, NBEAL2, NHP2, NOP10, PALB2, PUS1, RAD51C, RBM8A, RMRP, RPL11, RPL27, RPL35A, RPL5, RPS10, RPS19, RPS24, RPS27, RPS29, RPS7, RTEL1, RUNX1, S26, SBDS, SEC23B, SLC19A2, SLC25A38, SLC37A4, SMARCAL1, SRP72, TERC, TERT, TINF2, USB1, WAS, WRAP53, XRCC2	ABCB7, AK2, ALAS2, ANKRD26, BRCA2, BTHS, CDAN1, CTC1, CXCR4, DKC1, ELANE, ERCC1, ERCC4, FAAP95, FANCA, FANCC, FANCD2, FANCE, FANCF, FANCG, FANCI, FANCJ, FANCL, FANCM, FANCP, G6PC3, GATA1, GATA2, GFI1, GLRX5, GP1BA, HAX1, HOXA11, ITGAM, KLF1, LIG4, MASTL, MPL, MYH9, NBEAL2, NHP2, NOP10, PALB2, PARN, PUS1, RAD51C, RBM8A, RMRP, RPL11, RPL26, RPL27, RPL35A, RPL5, RPS10, RPS19, RPS24, RPS27, RPS29, RPS7, RTEL1, RUNX1, S26, SBDS, SEC23B, SLC19A2, SLC25A38, SLC37A4, SMARCAL1, SRP72, TERC, TERT, TINF2, USB1, VPS45, WAS, WRAP53, XRCC2	ABCB7, ABCG5, ABCG8, ACBD5, ACD, ACTN1, AK2 ALAS2, ANKRD26, AP3B1 ATR, ATRIP, BRAF, BRCA1 BRCA2, BTHS, C15ORF41, CBL CDAN1, CDC25C, CENPJ, CEP152, CEP63, CTC1, CXCR4, CYCS, DKC1, DNM2, ELANE, EPOR, ERCC1, ERCC4, ERCC6L2, ETV6, EXOC3L2, FAAP95, FANCA, FANCC, FANCD2, FANCE, FANCF, FANCG, FANCI, FANCJ, FANCL, FANCM, FANCP, FLNA, FYB, G6PC3, GATA1, GATA2, GCSFR, GFI1, GFI1B, GLRX5, GP1BA, HAX1, HOXA11, ITGA2B, ITGB3 JAGN1, KIF23, KLF1, KRAS, LIG4, LZTR1, MAP2K1, MASTL, MPL, MYH9, MYRIP, MYSM1, NBEAL2, NHP2, NOP10, NRAS, NSUN2, PALB2, PARN, PGM3, PRKACG, PTPN11, PUS1, RAD51C, RAF1, RASA2, RBBP8, RBM8A, RECQL4, RFPL4A, RIT1 RMRP, RPL11, RPL26, RPL27, RPL31, RPL35A, RPL5, RPS10, RPS17, RPS19, RPS24, RPS27, RPS28, RPS29, RPS7, RTEL1, RUNX1, S26, SBDS, SBF2, SEC23B, SHOC2, SLC19A2, SLC25A38, SLC37A4, SMARCAL1, SOS1, SOS2, SRP72, STIM1, TCIRG1, TERC, TERT, THPO, TINF2, TP53, TRNT1, TSR2, TUBB1, UBE2T, USB1, VPS13B, VPS45, WAS, WRAP53, XRCC2, XRCC5, YARS2

Supplementary Table 2: Primer sets used in the study

Name	Sequence	Size (bp)
RPS19 Exon3 forward	CAAGACCCTTAAATCTCCCTCT	127 bp
RPS19 Exon3 reverse	ATGACGCCGTTTCTCTGA	
RPS19 Exon4 forward	CACTCAGGGACAAAGAGATCT	101 bp
RPS19 Exon4 reverse	GTATGGGCTTTGGAAATGCTT	
RPL5 Exon2 forward	GTGACAGTTGTCTGTTTACTCTTGA	450 bp
RPL5 Exon3 reverse	GCTTCCCAAGCACAATTTCT	
RPL5 Exon4 forward	CCTCTTTGACTTTTTAAGCACCTC	441 bp
RPL5 Exon4 reverse	GCAGAAAACTGGAAGCAAGC	
RPL5 Exon5 forward	TTCCAGATGTCAGTGGTCCTT	446 bp
RPL5 Exon5 reverse	CTAGGTTCCTCCTGCATGGT	
RPL5 Exon6 forward	TTGTTTCAAGACGGGACTGA	450 bp
RPL5 Exon6 reverse	GCTTGAAGTCTCCCACACTTG	
RPL5 Exon7 forward	ACCACACCTGGCCTGATTTT	449 bp
RPL5 Exon7 reverse	TCACGCAGAGCTAGTGATCG	
RPL11 Exon2 forward	GTGTATTGACTGCTGCTCTT	198 bp
RPL11 Exon2 reverse	CCTTGTGACTACTCACCTTT	
RPL11 Exon4 forward	TGAGCTATTAATAGTTACTTGGGG	283 bp
RPL11 Exon4 reverse	GTGAACATACCACATATGAAATAA	
GATA2 Exon1 forward	TCTTCTTCAATCACCTCGACT	195 bp
GATA2 Exon1 reverse	ATTCCTGCGGATCCTACAT	
GATA2 Exon3 forward	CTGGACTCCCTCCCGAGAA	279 bp
GATA2 Exon3 reverse	TTTCCCCTGTAATTAACCGCCA	
GATA2 Exon5 forward	CATCTGGTGATGGGACTATGAA	231 bp
GATA2 Exon5 reverse	CTTGCTCTTCTTGGACTTGTT	
FANCA Exon2 forward	GTGAATTGTGCTGTGATGGTTT	172 bp
FANCA Exon2 reverse	GACACCAGCTTCCTCTTACCT	
FANCA Exon5 forward	AAGCTGGTTGGGAAAATAGGA	232 bp
FANCA Exon5 reverse	TCAACAGAACATTGCCTGGA	
FANCA Exon6 forward	ACTCAGAATGCTGGGGGTTT	217 bp
FANCA Exon6 reverse	CCGTCTGATTCTGGGCTTT	
FOXP2 forward	TGCTAGAGGAGTGGGACAAGTA	140 bp
FOXP2 reverse	GAAGCAGGACTCTAAGTGCAGA	
RPL35A Exon2 forward	GCTTAAACGAGAGGGGACGA	528 bp
RPL35A Exon3 reverse	CAGAGCAAGACTCCGTTATA	

Supplementary Table 3: Data and analysis of NGS reads at the RPS19 gene region from Patient 1,2 and 3 are shown. The normalized ratio are about 0.33 (range between 0.30-0.40) with multiple fragment in the gene and the likelihood ratio (in the deletion panel) are more than 20 which calculated from dispersion and normalized ratio of CNV. Based on this data the sequence change was determined as a deletion.

Patient 1

Description	Chr	CDS	Length	Ratio	HMM Calls
RPS19	chr19		472	0.386	Deletion
RPS19	chr19	1	171	0.3408	Deletion
RPS19	chr19	2	201	0.3664	Deletion
RPS19	chr19	3	284	0.3327	Deletion
RPS19	chr19	4	155	0.3463	Deletion
RPS19	chr19	5	166	0.3288	Deletion

Patient 2

Description	Chr	CDS	Length	Ratio	HMM Calls
RPS19	chr19		472	0.3609	Deletion
RPS19	chr19	1	171	0.3649	Deletion
RPS19	chr19	2	201	0.2738	Deletion
RPS19	chr19	3	284	0.3498	Deletion
RPS19	chr19	4	155	0.3946	Deletion
RPS19	chr19	5	166	0.3342	Deletion

Patient 3

Description	Chr	CDS	Length	Ratio	HMM Calls
RPS19	chr19		472	0.386	Deletion
RPS19	chr19	1	171	0.3408	Deletion
RPS19	chr19	2	201	0.3664	Deletion
RPS19	chr19	3	284	0.3327	Deletion
RPS19	chr19	4	155	0.3463	Deletion
RPS19	chr19	5	166	0.3288	Deletion

Supplementary Table 4: Data and analysis of NGS reads at the RPS19 gene region from Patient 4 are shown. The normalized ratio are about 0.33 (range between 0.30-0.40) with multiple fragment in the gene and the likelihood ratio (in the deletion panel) are more than 20 which calculated from dispersion and normalized ratio of CNV. Based on this data the sequence change was determined as a deletion.

Description	Chr	CDS	Length	Ratio	HMM Calls
RPL5_PROMOTER	chr1		697	0.3365	Deletion
RPL5	chr1	1	1724	0.3538	Deletion
RPL5	chr1		254	0.2018	Deletion
RPL5	chr1	4	235	0.316	Deletion
RPL5	chr1	6	1124	0.2684	Deletion
RPL5	chr1	7	189	0.3445	Deletion

Supplementary Table 5: Data and analysis of NGS reads at the RPS19 gene region from Patient 5 are shown. The normalized ratio are about 0.33 (range between 0.30-0.40) with multiple fragment in the gene and the likelihood ratio (in the deletion panel) are more than 20 which calculated from dispersion and normalized ratio of CNV. Based on this data the sequence change was determined as a deletion.

Description	Chr	CDS	Length	Ratio	HMM Calls
RPL11_PROMOTER	chr1		698	0.3062	Deletion
RPL11	chr1	1	382	0.3375	Deletion
RPL11	chr1	2	554	0.3329	Deletion
RPL11	chr1	3	207	0.3345	Deletion
RPL11	chr1	4	836	0.3198	Deletion
RPL11	chr1	5	431	0.3143	Deletion
RPL11	chr1	6	182	0.3391	Deletion

Supplementary Table 6: Data and analysis of NGS reads at the RPS19 gene region from Patient 8 are shown. The normalized ratio are about 0.33 (range between 0.30-0.40) with multiple fragment in the gene and the likelihood ratio (in the deletion panel) are more than 20 which calculated from dispersion and normalized ratio of CNV. Based on this data the sequence change was determined as a deletion.

Description	Chr	CDS	Length	Ratio	HMM Calls
GATA2	chr3	5	1997	0.3314	Deletion
GATA2	chr3	4	360	0.3299	Deletion
GATA2	chr3	3	246	0.3201	Deletion
GATA2	chr3	2	742	0.3371	Deletion
GATA2	chr3	1	374	0.3266	Deletion
GATA2	chr3		311	0.3593	Deletion
GATA2	chr3		279	0.3177	Deletion
GATA2	chr3		449	0.3135	Deletion
GATA2	chr3		319	0.3124	Deletion
GATA2	chr3		389	0.3224	Deletion

Supplementary Table 7: Data and analysis of NGS reads at the RPS19 gene region from Patient 9 are shown. The normalized ratio are about 0.33 (range between 0.30-0.40) with multiple fragment in the gene and the likelihood ratio (in the deletion panel) are more than 20 which calculated from dispersion and normalized ratio of CNV. Based on this data the sequence change was determined as a deletion.

Description	Chr	CDS	Length	Ratio	HMM Calls
FANCA	chr16	5	331	0.0022	Deletion
FANCA	chr16	4	243	0.0022	Deletion
FANCA	chr16	3	194	0.0049	Deletion
FANCA	chr16	2	881	0.003	Deletion
FANCA_PROMOTER	chr16		1001	0.004	Deletion

Supplemental Figure 1: Un-cropped images of the gels in Figure 2

Un-cropped image of the gel in figure 2B

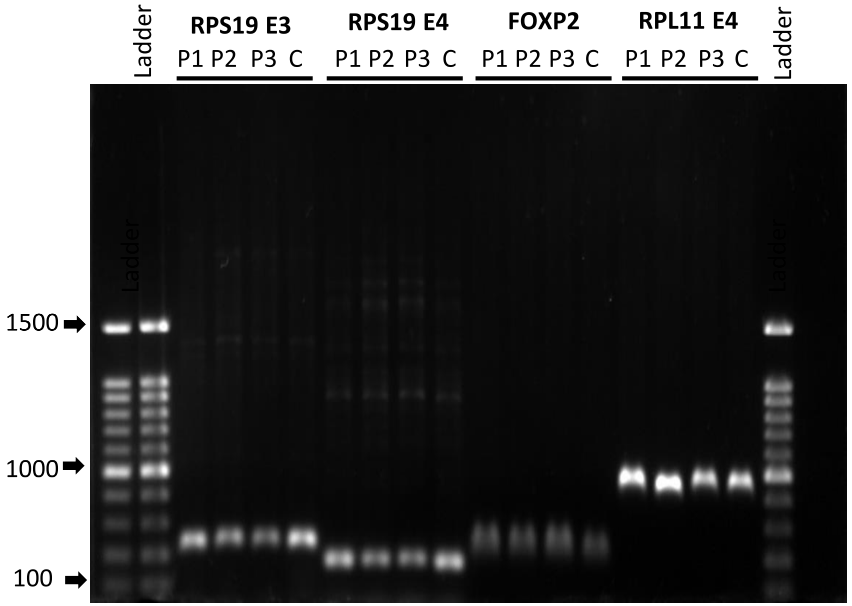

Un-cropped image of the gel in figure 2E

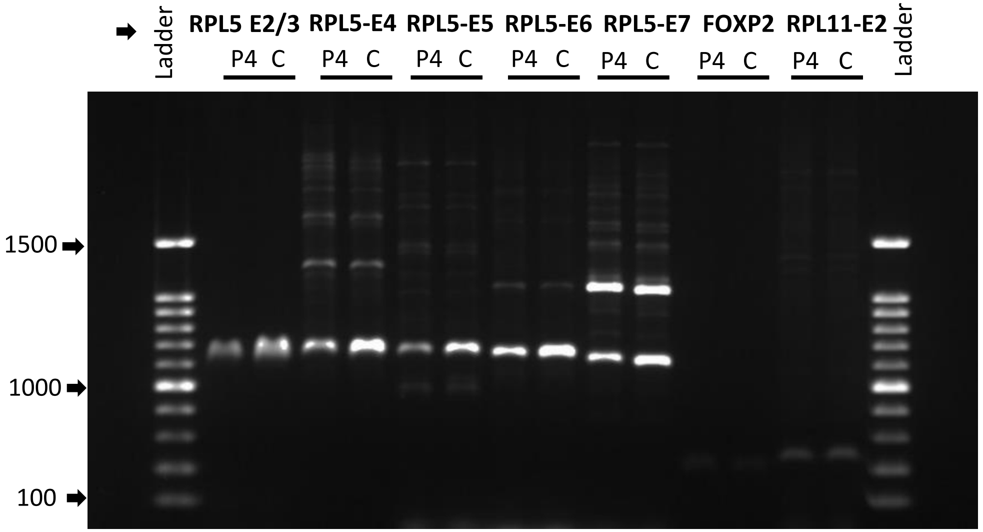

Un-cropped image of the gel in figure 2H

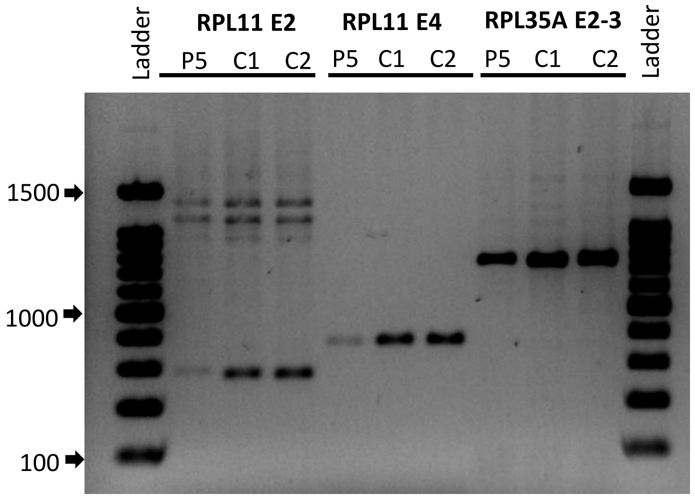

Supplemental Figure 2: Un-cropped image of the gel in Figure 3B

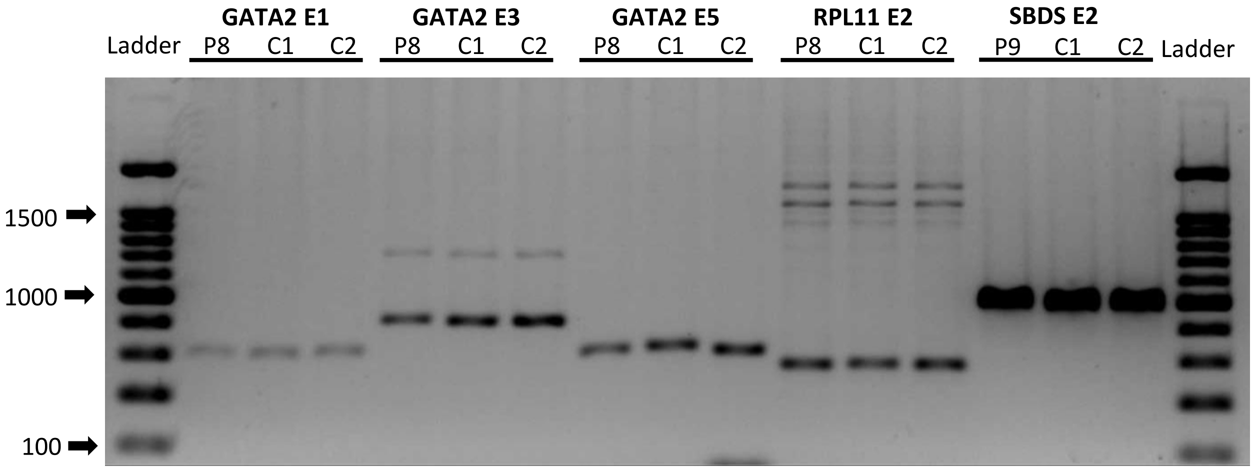

Supplemental Figure 3: Un-cropped image of the gel in Figure 4B

B

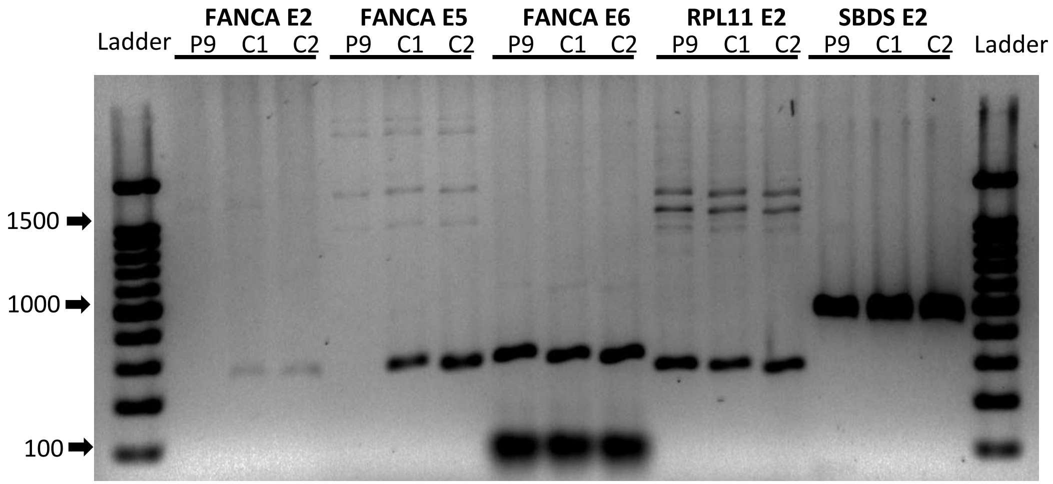

Supplement: Supplementary file 1 — Supplemental Material [file 41525_2019_104_MOESM1_ESM.pdf]
